# The metabolic-immune axis in MASH: is the macrophage signature the key to liver disease staging?

**DOI:** 10.1016/j.jlr.2026.101096

**Published:** 2026-07-01

**Authors:** Carlos Jose Pirola

**Affiliations:** 1 National Scientific and Technical Research Council, Buenos Aires, Argentina; 2 Systems Biology of Complex Diseases Department, Translational Research in Health Center, Maimonides University, Buenos Aires, Argentina

The clinical management of metabolic dysfunction-associated steatohepatitis (MASH) hinges on accurate staging, yet liver biopsy remains the invasive gold standard (1). Noninvasive biomarkers that capture the dynamic interplay between metabolic stress and immune remodeling are urgently needed. The advent of high-throughput omics techniques, even at the single-cell or nuclear level, has shed light on the role of immune cells in metabolic dysfunction-associated steatotic liver disease and its progression to MASH (2). However, the intricate cellular network involved remains elusive.

In this issue, Wang *et al.* (3) address this gap by leveraging single-nucleus RNA sequencing and bulk transcriptomics to identify a macrophage-specific gene signature that characterizes MASH with high diagnostic precision. The study moves beyond descriptive transcriptomics to delineate, a potentially causal link between macrophage activity and MASH, offering a robust framework for translational biomarker development (3).

The authors found five core macrophage-related differentially expressed genes—FERM domain containing 4B, protein tyrosine kinase 2 β, carboxypeptidase M (CPM), serine palmitoyltransferase long chain base subunit 2, and erythropoietin-producing hepatocellular carcinoma receptor B41-like 2—enriched within the M2 macrophage subset. They report that the diagnostic model achieves an area under the receiver operating characteristic curve exceeding 0.93 in independent validation samples. Through Mendelian randomization, the authors identified CPM as a protective causal factor, suggesting that elevated expression may represent a compensatory mechanism rather than a driver of pathology. Extensive validation across human cohorts, cholesterol-lard diet-fed mice, and polarized THP-1 cells underscores the biological relevance of these markers. The convergence of upregulated sphingolipid signaling and fatty acid metabolism pathways with TGF-β activation aligns with the profibrotic phenotype characteristic of advanced MASH.

Although the study presents a compelling diagnostic tool, critical scrutiny reveals nuances that merit consideration. The reliance on publicly available datasets, though robust, necessitates caution regarding cohort heterogeneity and batch effects inherent to multicenter transcriptomic data with modest sample size. The observed downregulation of FERM domain containing 4B and protein tyrosine kinase 2 β, alongside the upregulation of CPM, serine palmitoyltransferase long chain base subunit 2, and erythropoietin-producing hepatocellular carcinoma receptor B41-like 2, suggests complex regulatory networks that warrant further mechanistic dissection. Furthermore, while the diagnostic area under the receiver operating characteristic curve is impressive, the ultimate utility will depend on performance in longitudinal, ethnically diverse, and larger cohorts in a real-world clinical setting, as well as on comparison with existing noninvasive panels, such as enhanced liver fibrosis test or fibrosis-4 index. It is worth noting that simple liver steatosis and MASH may share some features; therefore, the power of these macrophage biomarkers to discriminate across the two disease grades remains to be demonstrated. The Mendelian randomization analysis strengthens causal inference for CPM (either directly or via the surrogate M2 macrophage polarization). Still, it cannot fully exclude horizontal pleiotropy or reverse causation in the context of metabolic diseases. As the authors point out, assigning causation in the absence of mechanistic experiments might be premature. Future work should assess whether this signature predicts progression to hepatocellular carcinoma, a risk inherent to the metabolic dysfunction-associated steatotic liver disease continuum.

The work of Wang *et al.* (3) intersects with emerging themes in liver immunology. Recent scRNA-seq atlases have emphasized the heterogeneity of Kupffer cells and infiltrating monocytes across disease stages (4). For instance, the identification of SPP1 and KLF2 as prognostic markers in chronic liver disease progression highlights the dynamic nature of macrophage transcriptional programs (4). Similarly, etiology-stratified analyses reveal that immune landscapes vary significantly across metabolic, alcoholic, and viral liver diseases, suggesting that macrophage biomarkers must be validated within specific etiologic contexts (5). Notably, the vision of M1/M2 polarized macrophages has been challenged by the putative existence of more complex states, such as lipid-associated macrophages in atherosclerosis or even the liver (6), in which CPM may be a marker of early differentiation to a “benign M2-like polarization” before lipid overload surpasses macrophage capacity (7).

Although the current study's focus on MASH provides a necessary foundation, the interplay between macrophage subsets and other immune cells, such as T follicular helper cells and regulatory T cells, remains a critical area for expansion (8). In addition, the link between mitochondrial dysfunction and immune remodeling, as seen in palmitoylation-based signatures, suggests that metabolic reprogramming in macrophages is a central node in MASH pathogenesis (9). The authors' emphasis on sphingolipid and fatty acid metabolism resonates with these findings, reinforcing the mitochondria-immune axis as a target for intervention. Interestingly, using the same snRNA-seq dataset (GSE212837), we demonstrated that MASH is characterized by high somatic heteroplasmy of oxidative phosphorylation chain components, also in macrophages, providing a solid genetic foundation for these deregulations (10).

The translation of this macrophage-specific signature from computational discovery to clinical application requires rigorous prospective validation. The development of liquid biopsy assays capable of detecting these markers in serum or plasma will be pivotal for real-world utility. Moreover, understanding the therapeutic potential of modulating CPM or the sphingolipid pathway could open new avenues for disease modification (Figure 1). As the field moves toward precision medicine in MASH, integrating single-cell resolution data with causal inference and multi-omics validation, as demonstrated by Wang *et al.* (3), will be essential. This new scenario not only advances the biomarker landscape but also reinforces the centrality of macrophage plasticity in MASH, urging the community to consider immune-metabolic crosstalk in both diagnostic and therapeutic strategies, as it has already been recognized in other cardiometabolic diseases.

**Fig. 1:**
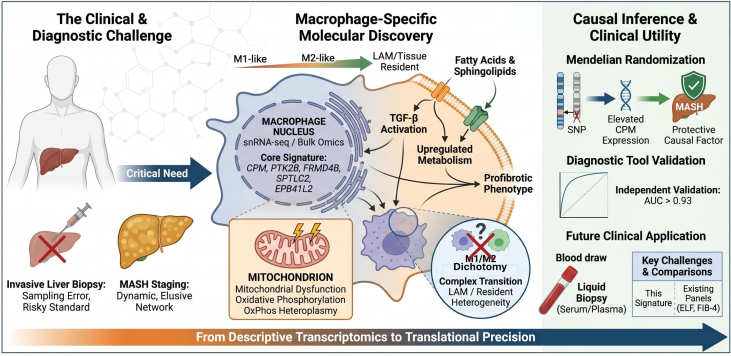
The evolving paradigm of macrophage-centered MASH diagnostics. Macrophage plasticity, metabolic stress, mitochondrial heteroplasmy, and TGF-β signaling converge to develop MASH. This signature (CPM, PTK2B, FRMD4B, SPTLC2, and EPB41L2) may move beyond mere description to offer causally inferable, high-precision noninvasive biomarkers for disease staging. MASH, metabolic dysfunction-associated steatohepatitis; TGF-β, transforming growth factor beta; CPM, carboxypeptidase M; PTK2B, protein tyrosine kinase 2 β; FRMD4B, FERM domain containing 4B; SPTLC2, serine palmitoyltransferase long chain base subunit 2; EPB41L2, erythropoietin-producing hepatocellular carcinoma receptor B41-like 2; LAM, lipid-associated macrophages; ELF, enhanced liver fibrosis test; and FIB-4, fibrosis-4 index.

## Conflict of Interest

The author declares that they have no conflicts of interest with the contents of this article.
